# Heat Sensitivity of *w*Mel *Wolbachia* during *Aedes aegypti* Development

**DOI:** 10.1371/journal.pntd.0004873

**Published:** 2016-07-26

**Authors:** Jill N. Ulrich, John C. Beier, Gregor J. Devine, Leon E. Hugo

**Affiliations:** 1 QIMR Berghofer Medical Research Institute, Royal Brisbane Hospital, Brisbane, Australia; 2 Leonard and Jayne Abess Center for Ecosystem Science and Policy, University of Miami, Coral Gables, Florida, United States of America; 3 Department of Public Health Sciences, University of Miami Miller School of Medicine, Miami, Florida, United States of America; Mahidol University, THAILAND

## Abstract

The *w*Mel strain of *Wolbachia* bacteria is known to prevent dengue and Zika virus transmission in the mosquito vector *Aedes aegypti*. Accordingly, the release of *w*Mel-infected *A*. *aegypti* in endemic regions has been recommended by the World Health Organization as a potential strategy for controlling dengue and Zika outbreaks. However, the utility of this approach could be limited if high temperatures in the aquatic habitats where *A*. *aegypti* develop are detrimental to *Wolbachia*. We exposed *w*Mel-infected *A*. *aegypti* eggs and larvae to fluctuating daily temperatures of 30–40°C for three, five, or seven days during their development. We found that *Wolbachia* levels in females emerging from heat treatments were significantly lower than in the controls that had developed at 20–30°C. Notably, seven days of high temperatures starting at the egg stage reduced *Wolbachia* levels in emerging females to less than 0.1% of the *w*Mel control levels. However, after adult females returned to 20–30°C for 4–7 days, they experienced differing degrees of *Wolbachia* recovery. Our findings suggest that the spread of *Wolbachia* in wild *A*. *aegypti* populations and any consequent protection from dengue and Zika viruses might be limited in ecosystems that experience periods of extreme heat, but *Wolbachia* levels recover partially after temperatures return to normal.

## Author Summary

The proposed arbovirus biocontrol strategy of releasing mosquitoes infected with the *w*Mel strain of *Wolbachia* bacteria promises to reduce the transmission of dengue and Zika viruses, but its utility in the field may be limited by the local environment. We show that when *Aedes aegypti* infected with *w*Mel experience high temperatures during egg and larval development, they have lower *Wolbachia* levels as emerging adults. High temperatures starting at the egg stage and lasting for seven days reduce *Wolbachia* levels in emerging females to less than 0.1% of control levels. However, partial recovery of *Wolbachia* occurs by 4–7 days of age. The spread of *Wolbachia* in wild *A*. *aegypti* populations and any resulting impacts on dengue and Zika transmission could be limited by periods of extreme heat, but *Wolbachia* levels may subsequently recover.

## Introduction

Mosquito-borne arboviruses are a growing public health threat. The alarming geographic spread and costly health burden of dengue fever have led the World Health Organization (WHO) to deem it “the most important mosquito-borne viral disease in the world.” Over the last 50 years, the incidence of dengue cases has increased 30-fold [1]. Now more than 100 countries have endemic dengue and over 40% of the world's population is at risk [2]. Zika virus, which recently caused a surge of children born with microcephaly and other neurological disorders, was declared a Public Health Emergency of International Concern (PHEIC) by the WHO after it spread from Brazil to 26 other countries or territories in the Americas within one year [3, 4]. With no effective antiviral treatments in the arsenal and just one licensed dengue vaccine that is 65.6% effective for those 9 years or older, control of the mosquito vectors, *Aedes aegypti* and *Aedes albopictus*, is the most viable option for curbing transmission of these arboviruses [5–7]. However, in resource-limited cities with endemic dengue, vector control efforts are often only pursued in response to explosive epidemics [8, 9]. Failure to control these vectors in tropical urban environments is one of the major drivers of the growing incidence and geographic expansion of dengue and other mosquito-borne arboviruses [10].

Alarmingly, existing control options for *A*. *aegypti* are of little use in urban areas [9, 11]. Space spraying with ultra-low volume insecticides, including organophosphates and pyrethroids, has been used by many countries in the face of dengue outbreaks for the past 40 years despite limited evidence of its epidemiological benefits [10, 12, 13]. Vector densities inevitably recover after space spraying because ideal larval habitats for *A*. *aegypti* abound in cities—exposed water sources for drinking or washing and non-biodegradable trash that collects water [8]. Targeted spraying of potential larval development containers with residual insecticides [14, 15] and indoor residual spraying targeting adult mosquitoes [16] in combination can substantially reduce local dengue incidence, but only if high coverage is achieved [17, 18]. For countries faced with nearly ubiquitous breeding of *A*. *aegypti* in their sprawling cities, the comprehensive spraying required to stop transmission is unrealistic [19]. Consequently, there is no urban setting in which vector control has completely eliminated dengue virus (DENV) transmission or prevented dengue epidemics [13, 20].

A potential solution for DENV and Zika virus (ZIKV) transmission involves releasing *A*. *aegypti* infected with *Wolbachia*, a common bacterium infecting the reproductive systems of many insects [21–24]. The fitness effects of *Wolbachia* on insect hosts are strain specific, ranging from life-shortening to pathogen-blocking phenotypes [25, 26]. The pathogen-blocking properties of some strains of *Wolbachia* have led researchers to characterize them further and transfer them into vector species for potential use in vector-borne disease control. The *w*Mel strain of *Wolbachia* shows the most promise currently, as it blocks DENV and ZIKV transmission by the mosquito, raising the possibility of disrupting dengue and Zika transmission cycles [27–30]. The *w*Mel strain was transinfected from *Drosophila melanogaster* into *A*. *aegypti*, and *w*Mel-infected *A*. *aegypti* have been released at sites in Australia, Vietnam, Brazil, Indonesia, and Colombia [31]. The success of each *Wolbachia* strain in invading insect populations is determined by the net fitness effect of the strain coupled with the extent to which it manipulates host reproduction [32]. One key mechanism of reproductive manipulation is cytoplasmic incompatibility (CI). When a strain causes complete CI, *Wolbachia*-infected females can mate successfully with *Wolbachia*-infected males, while uninfected females cannot [32]. The *w*Mel *Wolbachia* strain causes complete CI and has had some success in invading wild *A*. *aegypti* populations [28, 33]. However, the prevalence of *w*Mel *Wolbachia* must remain high in the *A*. *aegypti* population in order for *w*Mel to reliably and substantially reduce the capacity of the mosquito population to transmit pathogens [32, 34]. Protection against DENV in field-collected *w*Mel *A*. *aegypti* is similar to that observed in the original transinfected *w*Mel line [35], indicating that this strategy might be used to reduce dengue transmission in endemic areas [28, 29, 36]. Recently the WHO recommended the use of *Wolbachia* for dengue and Zika control [4], although there is currently insufficient epidemiological evidence to know if the approach is effective. It is also unknown whether the prevalence of *w*Mel-infected *A*. *aegypti* and the *wMel Wolbachia* levels within individual mosquitoes will remain high enough to prevent DENV and ZIKV transmission in all environments.

The levels of *w*Mel *Wolbachia* load throughout the various stages of the *A*. *aegypti* lifespan have not been described, as most studies have focused on population dynamics and fitness effects of *w*Mel *Wolbachia* after adult emergence [28, 33, 35, 37–40]. The early stages of development comprise a sensitive period during the *A*. *aegypti* lifespan; immature forms are confined to their aquatic habitats, whereas adults can seek out favorable microclimates to increase their chances of survival [41–43]. Immature *A*. *aegypti* develop in containers in the domestic environment that hold water, including flower pots, tanks, and drums as well as bottles, cans, and automobile tires [8, 44]. These containers sometimes hold as little as 5 mL of water [45]. Female *A*. *aegypti* preferentially lay their eggs in shaded containers, but it is not uncommon to find immatures in containers fully exposed to the sun [46, 47]. Although comprehensive temperature measurements in sun-exposed containers have not been carried out, lab-reared *A*. *aegypti* larvae can tolerate aquatic temperatures as high as 43°C if they are pre-exposed to high but sublethal temperatures [48]. The ability of *w*Mel *Wolbachia* to tolerate the same elevated temperatures as immature *A*. *aegypti* has not been investigated.

The heat sensitivity of *Wolbachia* with respect to its hosts has been characterized in other arthropods. Exposure to high temperatures during development cured the *Wolbachia* infections of two-spotted spider mites *Tetranychus urticae* [49], *Tribolium* flour beetles [50], and *Drosophila* spp. [51–54]. In the mosquito *Aedes scutellaris*, the reproductive effect of CI caused by *Wolbachia* was lost when larvae were reared at 32.5°C, but it was unknown whether the loss of *Wolbachia* or host expression of heat-shock proteins was responsible [55–57]. In *A*. *albopictus* all life stages maintained at 37°C had a lower levels of *Wolbachia* than those reared at 25°C, indicating that high temperatures may reduce *Wolbachia* levels in mosquito hosts [58].

Reduced *Wolbachia* levels in response to high temperatures during larval development could represent a barrier to the spread of *w*Mel *Wolbachia* in *A*. *aegypti* populations if fundamental drive mechanisms such as maternal transmission and CI are affected. Because only *Wolbachia*-infected females produce viable offspring with *Wolbachia*-infected males, CI creates a selective pressure for the spread of *Wolbachia* [32]. The spread of *Wolbachia* in mosquito populations is crucial, because incomplete *w*Mel *Wolbachia* coverage in the *A*. *aegypti* population leaves the potential for DENV and ZIKV transmission. A recent study found geographical clusters of uninfected mosquitoes in a *w*Mel-infected *A*. *aegypti* release suburb of Cairns, Far North Queensland, Australia [59]. The incomplete *Wolbachia* coverage was suggested to be due to immigration of uninfected mosquitoes from outside the release area, cryptic breeding sites, or other environmental phenomena such as “larval curing” (loss of *Wolbachia* infection during larval development) [59]. However, the occurrence of larval curing in mosquitoes has been poorly defined to date. Specifically, little is known about the temperature thresholds for *Wolbachia* during mosquito development or whether any potential curing persists after temperatures return to normal. Understanding larval curing in *w*Mel-infected *A*. *aegypti* has important applications, as lower *Wolbachia* levels in adults might have downstream impacts on cytoplasmic incompatibility [60–67] (although in *D*. *simulans* between-strain differences in CI are not explained by *Wolbachia* density [68]), maternal transmission [69, 70], and pathogen inhibition [29, 68, 71–73].

We investigated the effects of high temperatures during egg and larval development on laboratory-reared *w*Mel-infected *A*. *aegypti* using fluctuating daily temperatures that simulate the real-world conditions of a heatwave in Cairns, Australia. Our results have implications for the projected spread of *w*Mel *Wolbachia* through *A*. *aegypti* populations and for the vector competence of *w*Mel-infected *A*. *aegypti* under different environmental conditions.

## Methods

### Ethics statement

Blood feeding of mosquito colonies using human volunteers was performed in accordance to the QIMR Berghofer Human Research Ethics Committee permit QIMR HREC361. Written informed consent was obtained from all volunteers who participated in the study.

### Mosquitoes

Mosquitoes were taken from a *Wolbachia*-free *A*. *aegypti* colony (“Cairns” line) started from eggs collected in Cairns, Australia, in January 2015 and from a colony of *w*Mel-infected *A*. *aegypti* (“*w*Mel” line) started from eggs collected in suburbs of Cairns in April 2015. The colonies were maintained in separate, identical climate-controlled rooms at 27 ± 1°C and 70 ± 10% relative humidity with a 12:12 hour light:dark cycle and crepuscular periods. Eggs were flooded in aged (≥ 48 h) tap water and allowed to hatch naturally. Larval stages were reared under a controlled density (< 200 larvae per tray) in trays with 3 L of aged tap water. Larvae were fed on ground TetraMin tropical fish food (Tetra, Germany). Pupae were transferred into cages measuring 40 × 40 × 30 cm for adult emergence. Colonies were maintained with a population size of > 500 individuals per generation. Adult mosquitoes received 10% sucrose solution *ad libitum*, and females were blood-fed on a human volunteer for 15 min every 7 d. The *w*Mel-infected *A*. *aegypti* colony was regularly screened for *Wolbachia* using PCR of the *wsp* gene from the time of establishment [74]. Prior to the start of the experiments, our screening showed that the colony was completely infected with *Wolbachia*.

For the experiments, eggs were collected from *A*. *aegypti w*Mel (F_16_ and F_17_ generations used) and *A*. *aegypti* Cairns (F_18_ and F_19_ generations) colonies at 8:30 A.M. following the first night of oviposition. Eggs were counted under a stereomicroscope at 23°C and were separated into batches of approximately 600 eggs. Each batch was placed inside a dry paper towel, which was folded and placed next to a damp paper towel inside an open plastic bag. Egg bags were placed inside their corresponding environmental chambers at the coldest point of the temperature cycles, which was 20°C for the control condition and 30°C for the treatment condition. Eggs were left to mature for 48 h, and then batches of approximately 150 eggs were flooded in 500 mL aged tap water in plastic trays (183 × 152 × 65 mm). Four replicate trays were used per treatment group. From the day of hatching until pupation, ground TetraMin tropical fish food (Tetra, Germany) was administered daily at the coldest point of the temperature cycles using the “medium” diet described by Hugo *et al*. [75]. Pupae were transferred into 1-L plastic containers with mesh tops, and emerging adults were given 10% sucrose solution *ad libitum*. Adult females were aspirated out at 0–2 days post-emergence and at 4–7 days post-emergence. They were frozen at -20°C until processing.

### Environmental treatments

**Fig 1:**
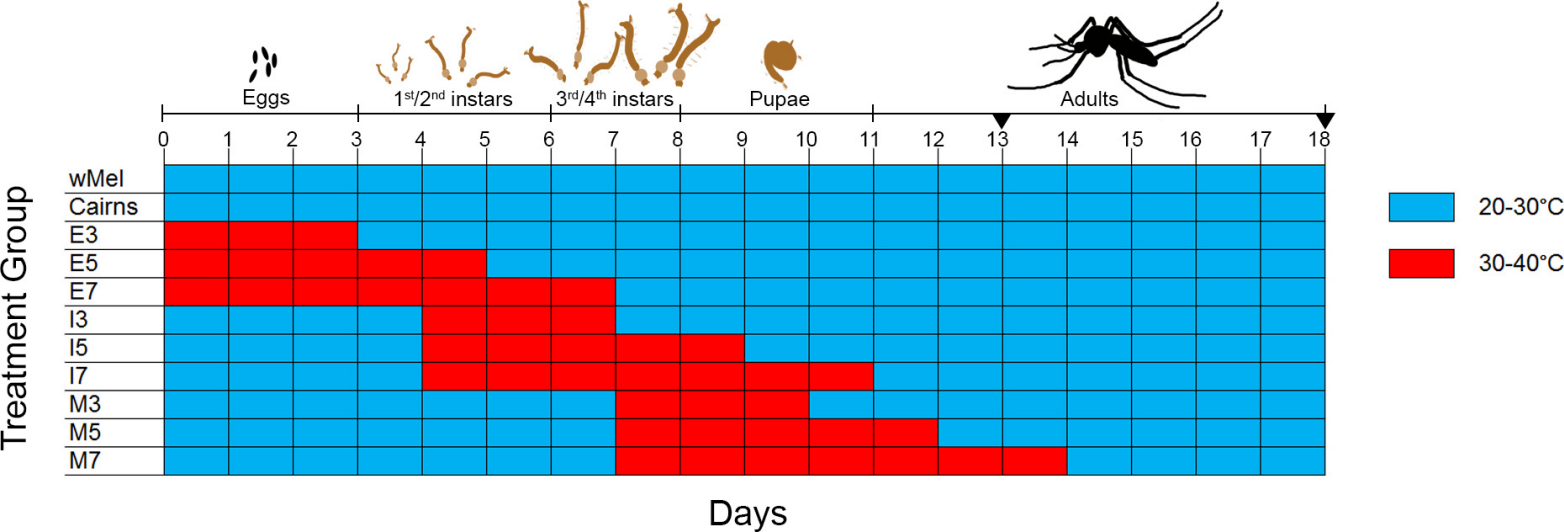
Experimental design. All treatment groups (rows) were transferred to environmental chambers as eggs within 15 hours of being laid, and all developed into adults by the end of the experiment. “*w*Mel” denotes the *w*Mel-infected *A*. *aegypti* controls, and “Cairns” denotes the wildtype (*Wolbachia*-free) *A*. *aegypti* controls. For each of the other treatment groups, the letter represents the stage of heat onset, with “E” indicating embryogenesis, “I” indicating immature larvae (1^st^/2^nd^ instars), and “M” indicating more mature larvae (3^rd^/4^th^ instars). The number represents the number of days the group remained in the high temperature treatment before returning to control temperatures (three days, five days, or seven days). Moving across each row, the cells track the days for each treatment group, with blue cells representing days spent in the control chamber and red cells representing days in the high temperatures chamber. Inverted triangles represent adult collection time points at 0–2 days and 4–7 days after emergence.

We tested the effect of high temperatures during egg and larval development on *Wolbachia* levels in *A*. *aegypti w*Mel adult females in two replicate experiments: Each replicate experiment compared various heatwave temperature regimes applied during particular periods of immature mosquito development that varied in duration and stage of onset. The temperature profiles we used simulated observed temperatures during average and extreme conditions in Cairns, Queensland. The Australian Bureau of Meteorology defines a heatwave as “a period of at least three days where the combined effect of excess heat and heat stress is unusual with respect to the local climate” [76]. We designed our treatment temperature profile to surpass the severe daily mean temperature threshold of 30.4°C for Cairns, which is based on temperature data from 1958 to 2011 [76]. Both treatment and control temperature profiles followed a truncated sinusoidal progression during the day and exponential decrease at night, representing a profile of daily temperature variation [77]. The shapes of the profiles were the same for each condition, but the profile was raised or lowered to adjust the mean temperature (S1 Fig). Experiments were conducted in two environmental chambers (294-L Panasonic MLR-352H-PE and MLR-351H, Gunma, Japan). Nine treatment groups were exposed to fluctuating heatwave temperatures between 30°C and 40°C for varying durations beginning at various life stages. Controls consisted of *w*Mel *A*. *aegypti* and wildtype Cairns *A*. *aegypti* exposed to diurnal temperature fluctuations between 20°C and 30°C. Transfers between environmental chambers were made at the coldest point of the temperature cycles (20°C for the control condition and 30°C for the treatment condition) in order to minimize the likelihood of heat shock. As illustrated in Fig 1, treatment groups exposed to high temperatures beginning from early embryogenesis (eggs at ≤ 15 hours post-oviposition) lasting three, five, or seven days are denoted by “E3,” “E5,” and “E7.” Groups exposed to high temperatures beginning at the immature larval stages (1^st^/2^nd^ instars) lasting three, five, or seven days are denoted by “I3,” “I5,” and “I7.” Groups exposed to high temperatures beginning at more mature larval stages (3^rd^/4^th^ instars) lasting three, five, or seven days are denoted by “M3,” “M5,” and “M7.” Prior to the two studies, a pilot study was conducted to determine differences in means for a range of onsets and durations (S2 Fig).

Data loggers, both factory installed and independent HOBO data loggers (Onset, Cape Cod, MA), recorded light intensity and temperature variation. Actual water temperatures in the control chamber were within 1.00°C of the programmed air temperature throughout the duration of the experiments. This was also the case in the treatment chamber, except during the coldest periods, when water temperature was as much as 2.93°C lower than the programmed air temperature.

### *Wolbachia* density in adult females

*Wolbachia* densities within individual adult females were determined by quantitative PCR. The head was removed from each frozen adult female before DNA extraction. Genomic DNA was extracted using QuickExtract DNA Extraction Solution (Epicentre Technologies Corporation) as per the manufacturer’s instructions and was diluted 1:10 in purified water. Multiplex qPCR was performed, amplifying the target *Wolbachia*-specific *wsp* gene and the somatic *Actin5c* gene, which acted as a reference gene to standardize for mosquito body size (*wsp* F: 5´–CATTGGTGTTGGTGTTGGTG–3´, R: 5´–ACACCAGCTTTTACTTGACCAG–3´, *Actin5c* F: 5´–GACGAAGAAGTTGCTGCTCTGGTTG–3´, R: 5´–TGAGGATACCACGCTTGCTCTGC–3´) (full methods in S1 Appendix) [73, 78, 79]. Quantification cycles (C_q_) were normalized by taking into consideration the different amplification efficiencies of the *wsp* and *Actin5c* genes, and *Wolbachia* to host genome ratios were calculated using Q-Gene [80].

### *Wolbachia* visualization in mosquito ovaries

Fluorescence *in situ* hybridization (FISH) was carried out using a *Wolbachia*-specific 16S rRNA probe [29]. Three freshly collected adult females (legs and wings removed) from each treatment group were fixed in 4% paraformaldehyde in 0.1 M phosphate buffer overnight and were transferred to 70% ethanol. Bodies were embedded in paraffin wax and sectioned with a microtome. Slides were dewaxed with two successive xylene washes for 10 min, two successive 5-min washes with 100% ethanol, and two successive 5-min washes in 95% ethanol. Slides were hybridized with the *Wolbachia*-specific *W2* probe (5´–CTTCTGTGAGTACCGTCATTATC–3´) [29] conjugated on the 5´ end to the fluorescent probe Alexa Fluor 488 (Molecular Probes, Inc). Slides were left in a dark humidity chamber at 37°C overnight and washed briefly in 1× saline sodium citrate (SSC) buffer + 10 mM dithiothreitol (DTT) at room temperature, then two 15-min washes in 1× SSC + 10 mM DTT at 55°C, two 15-min washes in 0.5× SSC at 55°C, a 10-min wash in 0.5× SSC + 10 mM DTT + 4',6-Diamidino-2-phenylindole (DAPI) (0.01 mg/50 mL) at room temperature, and then a final 10-min wash in 0.5× SSC + 10mM DTT at room temperature. Slides were washed briefly with distilled water and mounted with Vectashield Hard Set mounting medium (Vector Laboratories, Burlingame, CA). Slides were allowed to dry in a refrigerator overnight. Images from all sections were captured with a DeltaVision Core Deconvolution Microscope (GE) using identical acquisition settings (S2 Appendix). Images were reformatted using SoftWorx (Enterprise Softworks (Pty) Ltd.) and were cropped and standardized for contrast using Adobe Photoshop CS6 (Adobe Systems, Inc.).

### Body size

To determine the effect of the heat treatments on adult body size, the left wing of six females from each treatment group was removed and dry mounted on a slide. The distance from the axial notch to the wing tip, excluding the fringe scales, was used as a proxy for body size [75, 81].

### Statistical analysis

All analyses were performed in R [82] and GraphPad Prism v. 6 (GraphPad Software, San Diego, California, USA). Normality and homogeneity of variances within treatments were tested using Shapiro-Wilk and Bartlett’s tests, respectively. Log_10_-transformed *Wolbachia* densities were used for all analyses. A two-way blocked analysis of variance (ANOVA) was performed to determine the effects of treatment and collection time point and their interaction on *Wolbachia* density. Replicate was included as a blocking factor to account for any variation between the two experiments. An analogous two-way blocked ANOVA was performed to determine the effects of treatment group and collection time point and their interaction on body size. Pair-wise *post-hoc* comparisons between treatments and controls and between collection time points were made for both ANOVAs, and *P* values were adjusted for multiple comparisons using Tukey’s honest significant difference test. Differences were considered significant if adjusted *P* values were < 0.05. A nonlinear regression was performed using ordinary least squares fit for each stage of onset at the two collection time points to determine relationships between the heat treatment duration and *Wolbachia* density. Sum of squares F-tests were used to determine significant differences in slopes and y-intercepts.

## Results

### *Wolbachia* density in adult females

**Fig 2:**
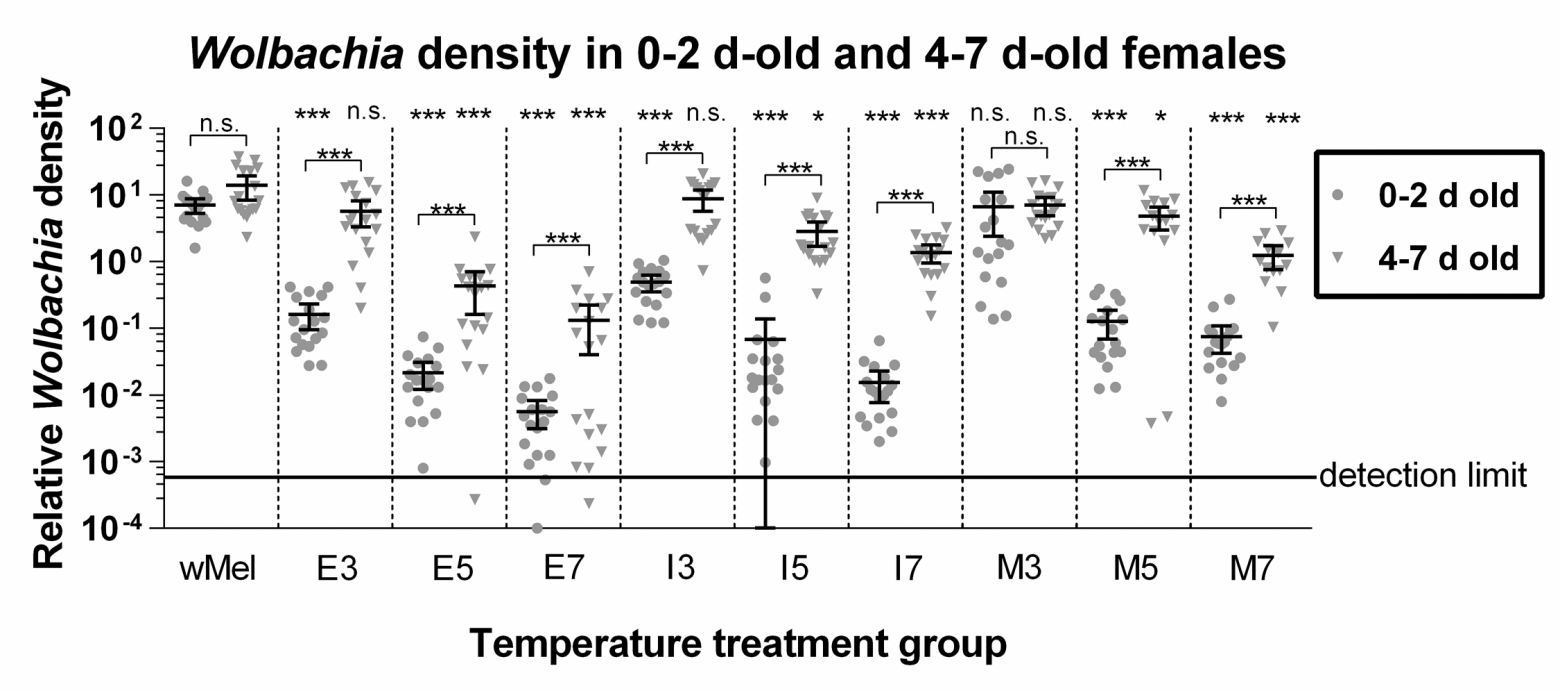
*Wolbachia* density by treatment group. *Wolbachia* densities in 0–2 d-old (circles) and 4–7 d-old (inverted triangles) female *w*Mel *A*. *aegypti* exposed to various temperature treatments. “*w*Mel” denotes the *w*Mel-infected *A*. *aegypti* controls that were not exposed to high temperatures, and for the other treatment groups the letter represents the stage of heat onset—“E” for embryogenesis, “I” for 1^st^/2^nd^ instars, and “M” for 3^rd^/4^th^ instars—and the number represents the number of days the group remained in the high temperature treatment. *Wolbachia* density was measured by qPCR of the *Wolbachia*-specific *wsp* gene and the somatic insect gene *Actin5c*. Displayed values are relative concentrations of *wsp* and *Actin5c* calculated in Q-Gene. The horizontal line at *y* = 10^−3.552^ represents the detection limit of *Wolbachia* by qPCR, which was established by the average C_q_ values for *Wolbachia*-free Cairns *A*. *aegypti* controls. Bars denote means bounded by their 95% confidence intervals. The lower 95% confidence limit for the 0–2 d-old I5 group (y = −0.009) is not shown because it cannot be represented on the log scale. The significance levels of differences between time points are indicated above brackets and between treatment groups and the *w*Mel controls at the top of the graph as *P* < 0.05 (*), *P* < 0.01 (**), *P* < 0.001 (***). Each point represents an individual mosquito.

We found significantly lower *Wolbachia* densities relative to *w*Mel controls in 0–2 d-old females emerging from eight of the nine treatments (Fig 2), with only the mature instar treatment lasting three days (M3) showing no significant reduction. *Wolbachia* levels in the 0–2 d-old females that were exposed to 30–40°C for seven days starting at the egg stage (E7) were less than 0.1% of *w*Mel control densities (Fig 2). Both treatment group and collection time point were significant predictors of *Wolbachia* density (*F*(10, 362) = 197.34, *MSE* = 55.24, *P* < 0.001 and *F*(1, 362) = 397.21, *MSE* = 111.20, *P* < 0.001, respectively). Compared with the 0–2 d adult collection time point, 4–7 d-old adult females in all treatment groups except the *w*Mel control group and the M3 group had higher *Wolbachia* levels, with adults from three-day treatments (E3, I3, and M3) showing *Wolbachia* densities that were not significantly different from *w*Mel-infected controls (Fig 2). The *Wolbachia* levels in 4–7 d-old adults from the six other treatments remained significantly lower than in *w*Mel-infected controls. There were inverse relationships between the duration of heat treatment and *Wolbachia* density and for all stages of onset; however, the relationships differed significantly both in their slopes and y-intercepts (*F*(5, 305) = 3.68, *P* = 0.003 and *F*(5, 305) = 2.79, *P* = 0.02, respectively). Duration of heat exposure had the greatest impact on *Wolbachia* density in emerging females when high temperatures began in the 3^rd^/4^th^ instar stages. At 4–7 days of age the impact of heat duration on density was most pronounced when high temperatures began at the egg stage.

### *Wolbachia* visualization in mosquito ovaries

**Fig 3:**
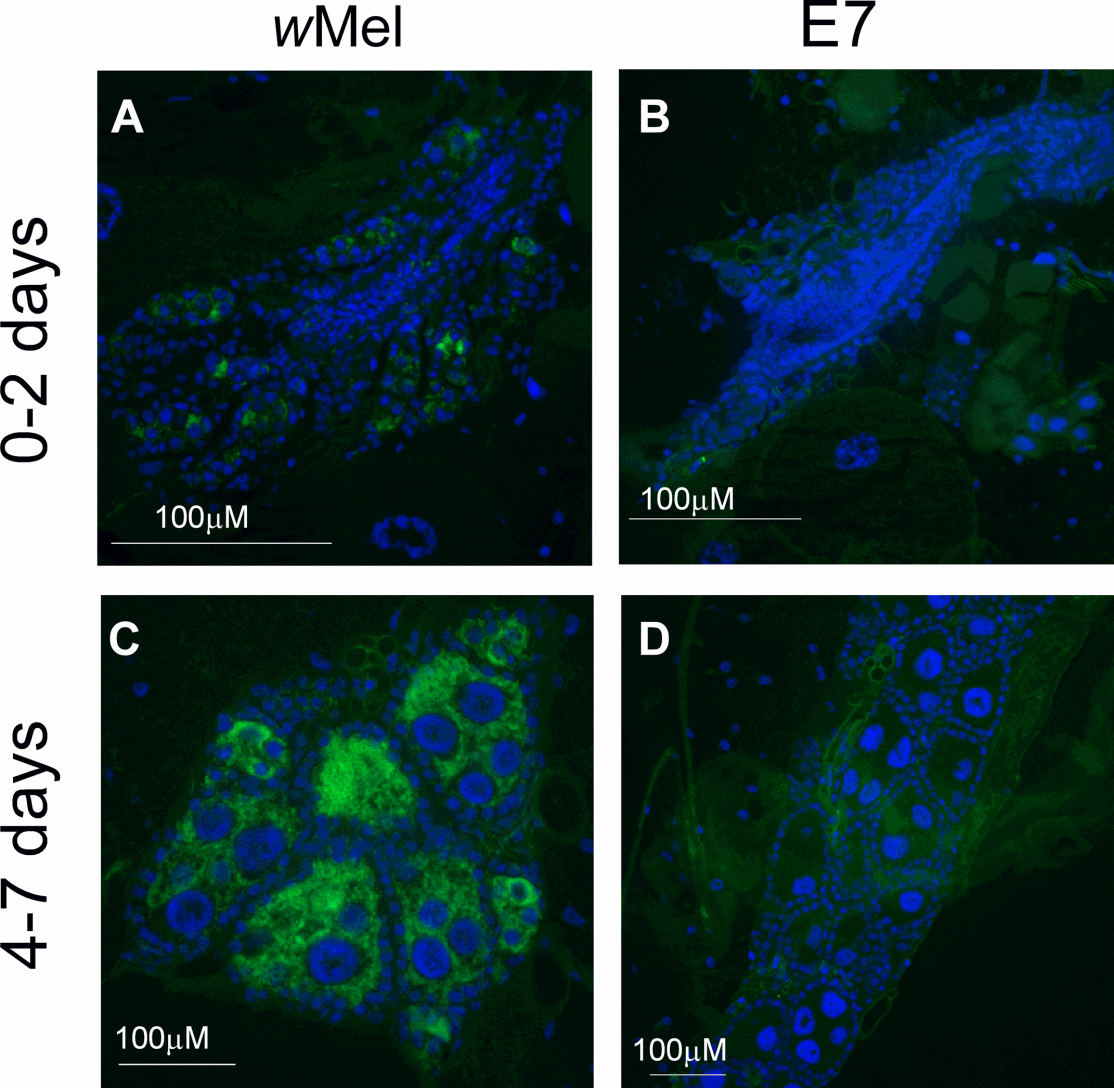
Visualization of *Wolbachia* in ovaries by FISH. Ovaries of *A*. *aegypti w*Mel females emerging from the control (A,C) and E7 (B,D) treatment groups, collected at 0–2 d (A, B) and 4–7 d (C, D) after emergence are shown. *Wolbachia* were stained with Alexa Fluor 488 (green) and cell nuclei with DAPI (blue).

We also investigated whether we could visualize reductions in *Wolbachia* levels in the ovaries of adult mosquitoes after exposure to high temperatures during development. Using FISH we visualized very low levels of *Wolbachia* in the ovaries of 0–2 d-old E7 females (Fig 3B). We also noticed that the E7 ovaries were much less developed than in controls, a possible consequence of the heat exposure. In 4–7 d-old E7 females (Fig 3D), *Wolbachia* remained at very low levels compared with 4–7 d-old *w*Mel-infected controls (Fig 3C).

### Body size

We found a significant effect of treatment group on wing length (*F*(10,71) = 13.70, *MSE =* 0.32, *P* < 0.001) and of the treatment group–collection time point interaction (*F*(9,71) = 2.81, *MSE =* 0.07, *P =* 0.007). Collection time point and replicate were not significant predictors (*F*(1, 71) = 0.22, *MSE* = 0.005, *P* = 0.64 and *F*(1, 71) = 1.21, *MSE* = 0.03, *P =* 0.27, respectively). Treatment groups E7, I5, I7, M3, M5, and M7 were all significantly smaller than *w*Mel controls (S3 Fig). There was no significant difference in wing length between *w*Mel controls and Cairns controls.

## Discussion

We found that when *A*. *aegypti* infected with the *w*Mel strain of *Wolbachia* were exposed to daily fluctuating temperatures of 30–40°C during early development, the emerging females had reduced *Wolbachia* levels compared with controls. The most affected group consisted of mosquitoes exposed to high temperatures starting at the egg stage and lasting for seven days (E7). In E7 emerging females, mean *Wolbachia* levels were less than 0.1% of the levels of *w*Mel controls. Loss of *Wolbachia* density from a subset of the mosquito population may be a concern for *Wolbachia-*based dengue and Zika control efforts in regions where the aquatic habitats of juvenile *A*. *aegypti* can reach extremely high temperatures. It has previously been shown that different *Wolbachia* strains attain different infection densities and that density is correlated with the level of virus inhibition [68, 71, 73, 78]. The relationship between *w*Mel density and DENV and ZIKV inhibition can be assumed from near complete blockage of these viruses in *Ae*. *aegypti* harboring dense *w*Mel infections [28, 30, 35], but the relationship has not been specifically defined. A recent study found that exposure of adult *w*Mel-infected *A*. *aegypti* to 28°C ± 4°C beginning at 5–8 d of adult age was associated with reduced *Wolbachia* densities; however, there was no interaction between the reduced densities and DENV infection, dissemination, or transmission [40]. Eggs and larvae exposed to high temperatures in our study produced adult *A*. *aegypti* with very low *Wolbachia* densities; therefore, the level of pathogen inhibition in adult mosquitoes that were subject to impacts of heat exposure during early development deserves investigation. The partial recovery of *Wolbachia* density by 4–7 days of age suggests that any impacts of heat exposure during mosquito development on subsequent virus inhibition may be attenuated with age.

This study is the first to investigate the duration and timing of heatwave conditions in relation to immature development of mosquitoes infected with *Wolbachia*. To achieve this we simulated normal and heatwave conditions based on temperature data from a city selected for *Wolbachia* biocontrol. We found an inverse relationship between the duration of heat exposure and *Wolbachia* density in adult females, raising the possibility that longer periods of heat might be capable of clearing *Wolbachia*. The slope of this relationship varied by the stage of heat onset and by the age of adult females collected. Duration of heat exposure had the greatest impact on *Wolbachia* density in emerging females when high temperatures began in the 3^rd^/4^th^ instar stages; however, the impact of heat duration on density at 4–7 days of age was most pronounced when high temperatures began at the egg stage. In addition to reducing bacterial densities, high temperatures resulted in smaller adult body sizes, with more prominent effects in the later stages of heat onset and the longer durations. This is likely due to the known inverse relationship between larval rearing temperature and adult body size [83]. We controlled for the effect of body size by standardizing *Wolbachia* density measurements with the host gene *Actin5c*.

Loss of *Wolbachia* density in response to heat has also been reported in *T*. *urticae* [49] *O*. *scapulalis* [84], *D*. *simulans* [54], *D*. *bifasciata* [53], *A*. *albopictus* [58], the predatory mite *Metaseiulus occidentalis* [85], and the wasp *Leptopilina heterotoma* [86]. The mechanism behind the loss of *Wolbachia* in response to high temperatures is not fully understood, but deformation of the *Wolbachia* cellular membrane could be a contributing factor [87]. Our FISH visualization confirms the loss of *Wolbachia* from the ovaries of mosquitoes exposed to high temperatures. Partial recovery of *Wolbachia* in the ovaries after the mosquito returns to normal temperatures suggests that *Wolbachia* replication continues even after the ovaries are fully developed. It is uncertain whether replication continues throughout the female lifespan and at what age *Wolbachia* densities would be restored to control levels in heat-exposed females.

Our results support the notion that *w*Mel has a more restricted thermotolerance than its mosquito host *A*. *aegypti*. Loss of thermotolerance in insect symbionts can be due to point mutations that occur as the symbiont co-evolves with the host [88]. In the case of the obligate symbiont of aphids *Buchnera aphidicola*, a point mutation affecting heat-shock protein transcription leads to death of the symbiont following a heat treatment [89]. Compared with other symbionts of insects, *w*Mel has experienced far less reductive evolution, as evidenced by its large genome with very high levels of repetitive DNA and mobile DNA elements [90]. Because of the low mutation rate of *w*Mel [90], loss of thermotolerance is less likely than for other symbionts [91]. If reductive evolution of *w*Mel does occur, then rearing *w*Mel-infected *A*. *aegypti* under constant temperatures in the lab might accelerate loss of *w*Mel thermotolerance. More studies are needed to understand the co-evolution of *w*Mel and *A*. *aegypti*.

*Wolbachia* may hold the potential to reduce and even eliminate dengue and Zika transmission in endemic areas. The advent of a promising control tool for dengue fever and Zika could not have come at a better time, as currently many tropical countries have no options to control the massive arbovirus outbreaks they experience. The strategy of releasing *w*Mel-infected *A*. *aegypti* is being tested in dengue-endemic regions around the globe, including Australia, Vietnam, Brazil, Indonesia, and Colombia [31], although substantial epidemiological data is still needed to assess the impacts on dengue and Zika transmission. The importance of measuring *Wolbachia* density in field trials, as opposed to presence or absence of *Wolbachia*, is highlighted by our results and other investigations [61, 64, 78]. We found that the high temperatures that *A*. *aegypti* may experience during early development can attenuate *w*Mel *Wolbachia* levels. Consequently, *w*Mel *Wolbachia* might be less effective as a dengue or Zika control strategy in regions experiencing periods of extreme heat. If the effectiveness is compromised, increased surveillance and supplementary mosquito control may be required in these regions. Further estimates of *Wolbachia* recovery rates after heat exposure are needed to understand the impacts on DENV and ZIKV inhibition and the spread of *w*Mel through naïve *A*. *aegypti* populations.

In summary, we showed that fluctuating daily temperatures of 30–40°C experienced during *w*Mel-infected *A*. *aegypti* egg and larval development significantly reduced *Wolbachia* levels in emerging adult females. However, *Wolbachia* recovered to differing degrees after adults returned to 20–30°C. These findings suggest that the effectiveness of *Wolbachia*-based arbovirus control might be compromised in ecosystems that experience periods of extreme heat, but given that *Wolbachia* levels partially recover after temperatures return to normal, any effects may be temporary. Greater understanding of environmental variables that affect *Wolbachia* can inform release site selection and help to better predict the impacts of *Wolbachia* on arbovirus transmission.

## Supporting Information

S1 Fig Water temperature fluctuations in environmental chambers. Control and treatment chambers are shown in blue and red, respectively. Data loggers were submerged in 500 mL aged tap water in trays (183 × 152 × 65 mm) and temperature was recorded every 30 min for the duration of both experiments. Bars denote means and standard errors over days logged.
(TIF)

S2 Fig Pilot study results. Log_10_-transformed relative *Wolbachia* densities in 0–2 d-old female *A*. *aegypti* in different treatment groups. Treatments not included in subsequent experiments include the pupae onset stage (P1, P3) and the one-day duration (I1, M1, P1). *Wolbachia* density was measured by qPCR of the *Wolbachia*-specific *wsp* gene and the somatic insect gene *Actin5c*. Displayed values are relative concentrations of *wsp* and *Actin5c* calculated in Q-Gene. Bars denote means bounded by their 95% confidence intervals. Significant differences between treatment groups and the *w*Mel controls are displayed as *P* < 0.05 (*), *P* < 0.01 (**), *P* < 0.001 (***). Each point represents an individual mosquito.
(TIF)

S3 Fig Wing length by treatment group. Significant differences between treatment groups and *w*Mel controls are indicated by asterisks, *P* <0.05 (*), *P* <0.01 (**), *P* <0.001 (***). Bars denote means bounded by their 95% confidence intervals. Each point represents an individual mosquito.
(TIF)

S1 Appendix qPCR conditions. The *Actin5c* gene was used to normalize *wsp* gene copies. qPCR reactions were performed in 10 μl total volume containing 5.0 μl Platinum SYBR Green qPCR SuperMix-UDG (Invitrogen), 1 μM of each primer, and 2 μL of DNA template. Cycling was performed using a RotorGene 6000 system (Corbett Research) with the following program: 95°C for 2 min, 50°C for 2 min, and 50 cyclic repeats of 95°C for 10 s, 52°C for 10 s, and 72°C for 20 s. This was followed by a standard melt analysis to confirm that only the expected product had been amplified. Quantification cycles (C_q_) values were calculated using the Comparative Quantification algorithm in the RotorGene 6000 software (Corbett Research). Repeat reactions were performed with samples for which the duplicate C_q_ values differed by more than 0.75.
(TIF)

S2 Appendix Microscopy methods. Images were captured with GE DeltaVision Core Deconvolution Microscope (GE) equipped with an Olympus X181 inverted microscope using an Olympus 20X/0.75 U Apo 340 lens or an Olympus 10X/0.40 D Plan Apo UV lens and a Photometrics Cool Snap HQ CCD camera. Images were acquired at a resolution of 1024 x 1024. DAPI excitation was 390/18nm and emission collection was 435/48 nm with 0.2 s exposure (5% ND filter). AlexaFluor 488 excitation was 475/28 nm and emission was 523/36 nm with a 0.15 s exposure (32% ND filter).
(TIF)

S1 Dataset *Wolbachia* density data from first replicate. (CSV)

S2 Dataset *Wolbachia* density data from second replicate. (CSV)

S3 Dataset Wing length data. (CSV)

## References

[1] World Health Organization. Global strategy for dengue prevention and control 2012–2020 Geneva: World Health Organization; 2012.

[2] World Health Organization. Factsheet: dengue and severe dengue September ed. Geneva: World Health Organization; 2013.

[3] ECDC. Rapid risk assessment: Zika virus epidemic in the Americas: potential association with microcephaly and Guillain-Barré syndrome, European Centre for Disease Prevention and Control, Stockholm 2015.

[4] WHO Vector Control Advisory Group. Mosquito (vector) control emergency response and preparedness for Zika virus Geneva: World Health Organization,. 2016 Available from: http://www.who.int/neglected_diseases/news/mosquito_vector_control_response/en/.

[5] Bhatt S, Gething PW, Brady OJ, Messina JP, Farlow AW, Moyes CL, et al The global distribution and burden of dengue. Nature. 2013. 10.1038/nature12060 PMC3651993 23563266

[6] McCall P, Kittayapong P. Control of dengue vectors: tools and strategies. Report of the Scientific Working Group on Dengue2006. p. 110–9.

[7] World Health Organization. Questions and Answers on Dengue Vaccines Geneva2016 [cited 2016]. Available from: http://www.who.int/immunization/research/development/dengue_q_and_a/en/.

[8] Monath TP. Dengue: the risk to developed and developing countries. Proc Natl Acad Sci USA. 1994;91(7):2395–400. 8146129 10.1073/pnas.91.7.2395 PMC43378

[9] Morrison AC, Zielinski-Gutierrez E, Scott TW, Rosenberg R. Defining challenges and proposing solutions for control of the virus vector *Aedes aegypti*. PLoS Med. 2008;5(3):e68 10.1371/journal.pmed.0050068 18351798 PMC2267811

[10] Gubler DJ. Prevention and control of *Aedes aegypti*-borne diseases: lessons learned from past successes and failures. Asia Pac J Mol Biol Biotechnol. 2011;19(3):111–4.

[11] Gubler DJ. Epidemic dengue/dengue hemorrhagic fever as a public health, social and economic problem in the 21st century. Trends Microbiol. 2002;10(2):100–3. 11827812 10.1016/s0966-842x(01)02288-0

[12] World Health Organization. Pesticides and their application: for the control of vectors and pests of public health importance Geneva: World Health Organization; 2006.

[13] Achee NL, Gould F, Perkins TA, Reiner RC Jr, Morrison AC, Ritchie SA, et al A critical assessment of vector control for dengue prevention. PLoS Negl Trop Dis. 2015;9(5). 10.1371/journal.pntd.0003655 PMC4423954 25951103

[14] Pettit WJ, Whelan PI, McDonnell J, Jacups SP. Efficacy of alpha-cypermethrin and lambda-cyhalothrin applications to prevent *Aedes* breeding in tires. J Am Mosq Control Assoc. 2010;26(4):387–97. 21290934 10.2987/09-5962.1

[15] Nguyen HT, Whelan PI, Shortus MS, Jacups SP. Evaluation of bifenthrin applications in tires to prevent *Aedes* mosquito breeding. J Am Mosq Control Assoc. 2009;25(1):74–82. 19432071 10.2987/08-5752.1

[16] Ritchie SA, Hanna JN, Hills SL, Piispanen JP, McBride WJH, Pyke A, et al Dengue control in north Queensland, Australia: case recognition and selective indoor residual spraying. Dengue Bull. 2002;26:7–13.

[17] Ritchie SA, Long S, Smith G, Pyke A, Knox TB. Entomological investigations in a focus of dengue transmission in Cairns, Queensland, Australia, by using the sticky ovitraps. J Med Entomol. 2004;41(1):1–4. 14989339 10.1603/0022-2585-41.1.1

[18] Vazquez-Prokopec GM, Kitron U, Montgomery B, Horne P, Ritchie SA. Quantifying the spatial dimension of dengue virus epidemic spread within a tropical urban environment. PLoS Negl Trop Dis. 2010;4(12):e920 10.1371/journal.pntd.0000920 21200419 PMC3006131

[19] Barreto ML, Teixeira MG, Bastos FI, Ximenes RA, Barata RB, Rodrigues LC. Successes and failures in the control of infectious diseases in Brazil: social and environmental context, policies, interventions, and research needs. Lancet. 2011;377(9780):1877–89. 10.1016/S0140-6736(11)60202-X 21561657

[20] Gubler DJ. Dengue, urbanization and globalization: the unholy trinity of the 21st century. Trop Med Health. 2011;39(4s):S3–S11. 10.2149/tmh.2011-S05 PMC3317603 22500131

[21] Bourtzis K, Dobson SL, Xi Z, Rasgon JL, Calvitti M, Moreira LA, et al Harnessing mosquito-*Wolbachia* symbiosis for vector and disease control. Acta Trop 2014;132:S150–S63. 10.1016/j.actatropica.2013.11.004 24252486

[22] Werren JH, Windsor DM. *Wolbachia* infection frequencies in insects: evidence of a global equilibrium? Proc R Soc Lond B Biol Sci. 2000;267(1450):1277–85. 10.1098/rspb.2000.1139 PMC1690679 10972121

[23] Jeyaprakash A, Hoy M. Long PCR improves *Wolbachia* DNA amplification: *wsp* sequences found in 76% of sixty-three arthropod species. Insect Mol Biol. 2000;9(4):393–405. 10971717 10.1046/j.1365-2583.2000.00203.x

[24] Zug R, Hammerstein P. Still a host of hosts for *Wolbachia*: analysis of recent data suggests that 40% of terrestrial arthropod species are infected. PloS One. 2012;7(6):e38544 10.1371/journal.pone.0038544 22685581 PMC3369835

[25] Hoffmann AA. Facilitating *Wolbachia* invasions. Aust Entomol. 2014;53(2):125–32.

[26] Bourtzis K, Lees RS, Hendrichs J, Vreysen MJ. More than one rabbit out of the hat: Radiation, transgenic and symbiont-based approaches for sustainable management of mosquito and tsetse fly populations. Acta Tropica. 2016. 10.1016/j.actatropica.2016.01.009 26774684

[27] Blagrove MS, Arias-Goeta C, Failloux A-B, Sinkins SP. *Wolbachia* strain *w*Mel induces cytoplasmic incompatibility and blocks dengue transmission in *Aedes albopictus*. Proc Natl Acad Sci USA. 2012;109(1):255–60. 10.1073/pnas.1112021108 22123944 PMC3252941

[28] Walker T, Johnson P, Moreira L, Iturbe-Ormaetxe I, Frentiu F, McMeniman C, et al The *w*Mel *Wolbachia* strain blocks dengue and invades caged *Aedes aegypti* populations. Nature. 2011;476(7361):450–3. 10.1038/nature10355 21866159

[29] Moreira LA, Iturbe-Ormaetxe I, Jeffery JA, Lu G, Pyke AT, Hedges LM, et al A *Wolbachia* symbiont in *Aedes aegypti* limits infection with dengue, chikungunya, and *Plasmodium*. Cell. 2009;139(7):1268–78. 10.1016/j.cell.2009.11.042 20064373

[30] Dutra HLC, Rocha MN, Dias FBS, Mansur SB, Caragata EP, Moreira LA. Wolbachia blocks currently circulating Zika virus isolates in Brazilian Aedes aegypti mosquitoes. Cell Host Microbe. 2016. 10.1016/j.chom.2016.04.021 PMC4906366 27156023

[31] Eliminate Dengue. Progress 2016 [cited 2016 1 March]. Available from: http://www.eliminatedengue.com/progress.

[32] Werren JH, Baldo L, Clark ME. *Wolbachia*: master manipulators of invertebrate biology. Nat Rev Microbiol. 2008;6(10):741–51. 10.1038/nrmicro1969 18794912

[33] Hoffmann A, Montgomery B, Popovici J, Iturbe-Ormaetxe I, Johnson P, Muzzi F, et al Successful establishment of *Wolbachia* in *Aedes* populations to suppress dengue transmission. Nature. 2011;476(7361):454–7. 10.1038/nature10356 21866160

[34] Bull JJ, Turelli M. *Wolbachia* versus dengue: evolutionary forecasts. Evol Med Public Health. 2013;2013(1):197–207. 10.1093/emph/eot018 24481199 PMC3847891

[35] Frentiu FD, Zakir T, Walker T, Popovici J, Pyke AT, van den Hurk A, et al Limited dengue virus replication in field-collected *Aedes aegypti* mosquitoes infected with *Wolbachia*. PLoS Negl Trop Dis. 2014;8(2):e2688 10.1371/journal.pntd.0002688 24587459 PMC3930499

[36] Bian G, Xu Y, Lu P, Xie Y, Xi Z. The endosymbiotic bacterium *Wolbachia* induces resistance to dengue virus in *Aedes aegypti*. PLoS Pathog. 2010;6(4):e1000833 10.1371/journal.ppat.1000833 20368968 PMC2848556

[37] Bian G, Joshi D, Dong Y, Lu P, Zhou G, Pan X, et al Wolbachia invades *Anopheles stephensi* populations and induces refractoriness to *Plasmodium* infection. Science. 2013;340(6133):748–51. 10.1126/science.1236192 23661760

[38] Van den Hurk F, Hall-Mendelin S, Pyke AT, Frentiu FD, McElroy K, Day A, et al Impact of *Wolbachia* on infection with chikungunya and yellow fever viruses in the mosquito vector *Aedes aegypti*. PLoS Negl Trop Dis. 2012;6(11). 10.1371/journal.pntd.0001892 PMC3486898 23133693

[39] Turley AP, Zalucki MP, O'Neill SL, McGraw EA. Transinfected *Wolbachia* have minimal effects on male reproductive success in *Aedes aegypti*. Parasit Vectors. 2013;6:36 10.1186/1756-3305-6-36 23399027 PMC3584945

[40] Yixin HY, Carrasco AM, Dong Y, Sgrò CM, McGraw EA. The effect of temperature on *Wolbachia*-mediated dengue virus blocking in *Aedes aegypti*. Am J Trop Med Hyg. 2016:15–0801. Epub Feb 8. 10.4269/ajtmh.15-0801 PMC4824223 26856916

[41] Reiter P. Oviposition, dispersal, and survival in *Aedes aegypti*: implications for the efficacy of control strategies. Vector Borne Zoonotic Dis. 2007;7(2):261–73. 17627447 10.1089/vbz.2006.0630

[42] Kessler S, Guerin PM. Responses of *Anopheles gambiae*, *Anopheles stephensi*, *Aedes aegypti*, and *Culex pipiens* mosquitoes (Diptera: Culicidae) to cool and humid refugium conditions. J Vector Ecol. 2008;33(1):145–9. 18697317 10.3376/1081-1710(2008)33[145:roagas]2.0.co;2

[43] Perich M, Davila G, Turner A, Garcia A, Nelson M. Behavior of resting *Aedes aegypti* (Culicidae: Diptera) and its relation to ultra-low volume adulticide efficacy in Panama City, Panama. J Med Entomol. 2000;37(4):541–6. 10916294 10.1603/0022-2585-37.4.541

[44] Chan K, Ho B, Chan Y. *Aedes aegypt*i (L.) and *Aedes albopictus* (Skuse) in Singapore City: 2. Larval habitats*. Bull World Health Org. 1971;44(5):629 5316746 PMC2427856

[45] Simard F, Nchoutpouen E, Toto JC, Fontenille D. Geographic distribution and breeding site preference of *Aedes albopictus* and *Aedes aegypti* (Diptera: Culicidae) in Cameroon, Central Africa. J Med Entomol. 2005;42(5):726–31. 16363155 10.1093/jmedent/42.5.726

[46] Tun-Lin W, Burkot T, Kay B. Effects of temperature and larval diet on development rates and survival of the dengue vector *Aedes aegypti* in north Queensland, Australia. Med Vet Entomol. 2000;14(1):31–7. 10759309 10.1046/j.1365-2915.2000.00207.x

[47] Vezzani D, Albicocco A. The effect of shade on the container index and pupal productivity of the mosquitoes *Aedes aegypti* and *Culex pipiens* breeding in artificial containers. Med Vet Entomol. 2009;23(1):78–84. 10.1111/j.1365-2915.2008.00783.x 19239617

[48] Patil NS, Lole KS, Deobagkar DN. Adaptive larval thermotolerance and induced cross-tolerance to propoxur insecticide in mosquitoes *Anopheles stephensi* and *Aedes aegypti*. Med Vet Entomol. 1996;10(3):277–82. 8887340 10.1111/j.1365-2915.1996.tb00743.x

[49] Van Opijnen T, Breeuwer J. High temperatures eliminate *Wolbachia*, a cytoplasmic incompatibility inducing endosymbiont, from the two-spotted spider mite. Exp Appl Acarol. 1999;23(11):871–81. 10668862 10.1023/a:1006363604916

[50] Stevens L. Environmental factors affecting reproductive incompatibility in flour beetles, genus *Tribolium*. J Invertebr Pathol. 1989;53(1):78–84. 2915149 10.1016/0022-2011(89)90076-1

[51] Hoffmann AA, Turelli M, Simmons GM. Unidirectional incompatibility between populations of *Drosophila simulans*. Evolution. 1986:692–701. 28556160 10.1111/j.1558-5646.1986.tb00531.x

[52] Hoffmann AA, Turelli M, Harshman LG. Factors affecting the distribution of cytoplasmic incompatibility in *Drosophila simulans*. Genetics. 1990;126(4):933–48. 2076821 10.1093/genetics/126.4.933 PMC1204290

[53] Hurst GD, Johnson AP, vd Schulenburg JHG, Fuyama Y. Male-killing *Wolbachia* in *Drosophila*: a temperature-sensitive trait with a threshold bacterial density. Genetics. 2000;156(2):699–709. 11014817 10.1093/genetics/156.2.699 PMC1461301

[54] Clancy DJ, Hoffmann AA. Environmental effects on cytoplasmic incompatibility and bacterial load in *Wolbachia*-infected *Drosophila simulans*. Entomol Exp Appl. 1998;86(1):13–24.

[55] Trpis M, Perrone J, Reissig M, Parker K. Control of cytoplasmic incompatibility in the *Aedes scutellaris* complex: incompatible crosses become compatible by treatment of larvae with heat or antibiotics. Journal of Heredity. 1981;72(5):313–7.

[56] Feder ME, Karr TL, Yang W, Hoekstra JM, James AC. Interaction of *Drosophila* and its endosymbiont *Wolbachia*: natural heat shock and the overcoming of sexual incompatibility. Am Zool. 1999;39(2):363–73.

[57] Xi Z, Gavotte L, Xie Y, Dobson SL. Genome-wide analysis of the interaction between the endosymbiotic bacterium *Wolbachia* and its *Drosophila* host. BMC Genomics. 2008;9(1):1. 18171476 10.1186/1471-2164-9-1 PMC2253531

[58] Wiwatanaratanabutr I, Kittayapong P. Effects of crowding and temperature on *Wolbachia* infection density among life cycle stages of *Aedes albopictus*. J Invertebr Pathol. 2009;102(3):220–4. 10.1016/j.jip.2009.08.009 19686755

[59] Hoffmann AA, Iturbe-Ormaetxe I, Callahan AG, Phillips BL, Billington K, Axford JK, et al Stability of the *w*Mel *Wolbachia* infection following invasion into *Aedes aegypti* populations. PLoS Negl Trop Dis. 2014;8(9):e3115 10.1371/journal.pntd.0003115 25211492 PMC4161343

[60] Boyle L, O'Neill SL, Robertson HM, Karr TL. Interspecific and intraspecific horizontal transfer of *Wolbachia* in *Drosophila*. Science. 1993;260(5115):1796–9. 8511587 10.1126/science.8511587

[61] Breeuwer J, Werren JH. Cytoplasmic incompatibility and bacterial density in *Nasonia vitripennis*. Genetics. 1993;135(2):565–74. 8244014 10.1093/genetics/135.2.565 PMC1205656

[62] Solignac M, Vautrin D, Rousset F. Widespread occurence of the proteobacteria *Wolbachia* and partial cytoplasmic incompatibility in *Drosophila melanogaster*. C R Acad Sci III. 1994;317(5):461–70.

[63] Mercot H, Llorente B, Jacques M, Atlan A, Montchamp-Moreau C. Variability within the Seychelles cytoplasmic incompatibility system in *Drosophila simulans*. Genetics. 1995;141(3):1015–23. 8582608 10.1093/genetics/141.3.1015 PMC1206825

[64] Sinkins S, Braig H, Oneill S. *Wolbachia pipientis*: bacterial density and unidirectional cytoplasmic incompatibility between infected populations of *Aedes albopictus*. Exp Parasitol. 1995;81(3):284–91. 7498425 10.1006/expr.1995.1119

[65] Bourtzis K, Nirgianaki A, Markakis G, Savakis C. *Wolbachia* infection and cytoplasmic incompatibility in *Drosophila* species. Genetics. 1996;144(3):1063–73. 8913750 10.1093/genetics/144.3.1063 PMC1207602

[66] Veneti Z, Clark ME, Zabalou S, Karr TL, Savakis C, Bourtzis K. Cytoplasmic incompatibility and sperm cyst infection in different *Drosophila*-*Wolbachia* associations. Genetics. 2003;164(2):545–52. 12807775 10.1093/genetics/164.2.545 PMC1462605

[67] Calvitti M, Marini F, Desiderio A, Puggioli A, Moretti R. Wolbachia density and cytoplasmic incompatibility in *Aedes albopictus*: Concerns with using artificial *Wolbachia* infection as a vector suppression tool. PloS One. 2015;10(3):e0121813 10.1371/journal.pone.0121813 25812130 PMC4374832

[68] Martinez J, Ok S, Smith S, Snoeck K, Day JP, Jiggins FM. Should symbionts be nice or selfish? Antiviral effects of Wolbachia are costly but reproductive parasitism is not. PLoS Pathog. 2015;11(7):e1005021 10.1371/journal.ppat.1005021 26132467 PMC4488530

[69] Dutton TJ, Sinkins SP. Strain-specific quantification of *Wolbachia* density in *Aedes albopictus* and effects of larval rearing conditions. Insect Mol Biol. 2004;13(3):317–22. 15157232 10.1111/j.0962-1075.2004.00490.x

[70] Veneti Z, Clark ME, Karr TL, Savakis C, Bourtzis K. Heads or tails: host-parasite interactions in the *Drosophila-Wolbachia* system. Appl Environ Microbiol. 2004;70(9):5366–72. 15345422 10.1128/AEM.70.9.5366-5372.2004 PMC520876

[71] Lu P, Bian G, Pan X, Xi Z. *Wolbachia* induces density-dependent inhibition to dengue virus in mosquito cells. PLoS Negl Trop Dis. 2012;6(7):e1754–e. 10.1371/journal.pntd.0001754 22848774 PMC3404113

[72] Frentiu FD, Robinson J, Young PR, McGraw EA, O’neill SL. *Wolbachia*-mediated resistance to dengue virus infection and death at the cellular level. PloS One. 2010;5(10):e13398 10.1371/journal.pone.0013398 20976219 PMC2955527

[73] Martinez J, Longdon B, Bauer S, Chan Y-S, Miller WJ, Bourtzis K, et al Symbionts commonly provide broad spectrum resistance to viruses in insects: a comparative analysis of *Wolbachia* strains. PLoS Pathog. 2014;10(9):e1004369 10.1371/journal.ppat.1004369 25233341 PMC4169468

[74] Braig H, Zhou W, Dobson S, O'Neill S. Cloning and characterization of a gene encoding the major surface protein of the bacterial endosymbiont *Wolbachia pipientis*. Journal of Bacteriology. 1998;180(9):2373–8. 9573188 10.1128/jb.180.9.2373-2378.1998 PMC107178

[75] Hugo LE, Kay BH, O'neill SL, Ryan PA. Investigation of environmental influences on a transcriptional assay for the prediction of age of *Aedes aegypti* (Diptera: Culicidae) mosquitoes. J Med Entomol. 2010;47(6):1044–52. 21175052 10.1603/me10030

[76] Nairn JR, Fawcett RG, Day KA. Defining heatwaves: heatwave defined as a heat-impact event servicing all community and business sectors in Australia: Centre for Australian Weather and Climate Research; 2013.

[77] Parton WJ, Logan JA. A model for diurnal variation in soil and air temperature. Agricultural Meteorology. 1981;23:205–16.

[78] Osborne SE, Iturbe-Ormaetxe I, Brownlie JC, O'Neill SL, Johnson KN. Antiviral protection and the importance of *Wolbachia* density and tissue tropism in *Drosophila simulans*. Appl Environ Microbiol. 2012;78(19):6922–9. 10.1128/AEM.01727-12 22843518 PMC3457512

[79] Osborne SE, Leong YS, O’Neill SL, Johnson KN. Variation in antiviral protection mediated by different *Wolbachia* strains in *Drosophila simulans*. PLoS Pathog. 2009;5(11):e1000656–e. 10.1371/journal.ppat.1000656 19911047 PMC2768908

[80] Simon P. Q-Gene: processing quantitative real-time RT–PCR data. Bioinformatics. 2003;19(11):1439–40. 12874059 10.1093/bioinformatics/btg157

[81] Nasci RS. Relationship of wing length to adult dry weight in several mosquito species (Diptera: Culicidae). J Med Entomol. 1990;27:716–9. 2388250 10.1093/jmedent/27.4.716

[82] R Development Core Team. R: A language and environment for statistical computing. Vienna, Austria: R Foundation for Statistical Computing; 2010.

[83] Rueda L, Patel K, Axtell R, Stinner R. Temperature-dependent development and survival rates of *Culex quinquefasciatus* and *Aedes aegypti* (Diptera: Culicidae). J Med Entomol. 1990;27(5):892–8. 2231624 10.1093/jmedent/27.5.892

[84] Sugimoto TN, Kayukawa T, Matsuo T, Tsuchida T, Ishikawa Y. A short, high-temperature treatment of host larvae to analyze *Wolbachia*–host interactions in the moth *Ostrinia scapulalis*. J Insect Physiol. 2015;81:48–51. 10.1016/j.jinsphys.2015.06.016 26142572

[85] Jeyaprakash A, Hoy MA. Real-time PCR reveals endosymbiont titer fluctuations in *Metaseiulus occidentalis* (Acari: Phytoseiidae) colonies held at different temperatures. Fla Entomol. 2010;93(3):464–6.

[86] Mouton L, Henri H, Charif D, Boulétreau M, Vavre F. Interaction between host genotype and environmental conditions affects bacterial density in *Wolbachia* symbiosis. Biol Lett. 2007;3(2):210–3. 17251124 10.1098/rsbl.2006.0590 PMC2375926

[87] Zhukova M, Voronin D, Kiseleva E. High temperature initiates changes in *Wolbachia* ultrastructure in ovaries and early embryos of *Drosophila melanogaster*. Cell tissue biol. 2008;2(5):546–56. 19198544

[88] Zilber-Rosenberg I, Rosenberg E. Role of microorganisms in the evolution of animals and plants: the hologenome theory of evolution. FEMS Microbiol Rev. 2008;32(5):723–35. 10.1111/j.1574-6976.2008.00123.x 18549407

[89] Dunbar HE, Wilson AC, Ferguson NR, Moran NA. Aphid thermal tolerance is governed by a point mutation in bacterial symbionts. PLoS Biol. 2007;5(5):e96 17425405 10.1371/journal.pbio.0050096 PMC1847839

[90] Wu M, Sun LV, Vamathevan J, Riegler M, Deboy R, Brownlie JC, et al Phylogenomics of the reproductive parasite *Wolbachia pipientis w*Mel: a streamlined genome overrun by mobile genetic elements. PLoS Biol. 2004;2(3):E69–E. 15024419 10.1371/journal.pbio.0020069 PMC368164

[91] Moran NA, McCutcheon JP, Nakabachi A. Genomics and evolution of heritable bacterial symbionts. Annu Rev Genet. 2008;42:165–90. 10.1146/annurev.genet.41.110306.130119 18983256

