# Supplementary figures and images for: Heat Sensitivity of wMel Wolbachia during Aedes aegypti Development
Source: PLoS Negl Trop Dis. 2016 Jul 26;10(7):e0004873. doi: 10.1371/journal.pntd.0004873 (PMC4961373; doi:10.1371/journal.pntd.0004873)

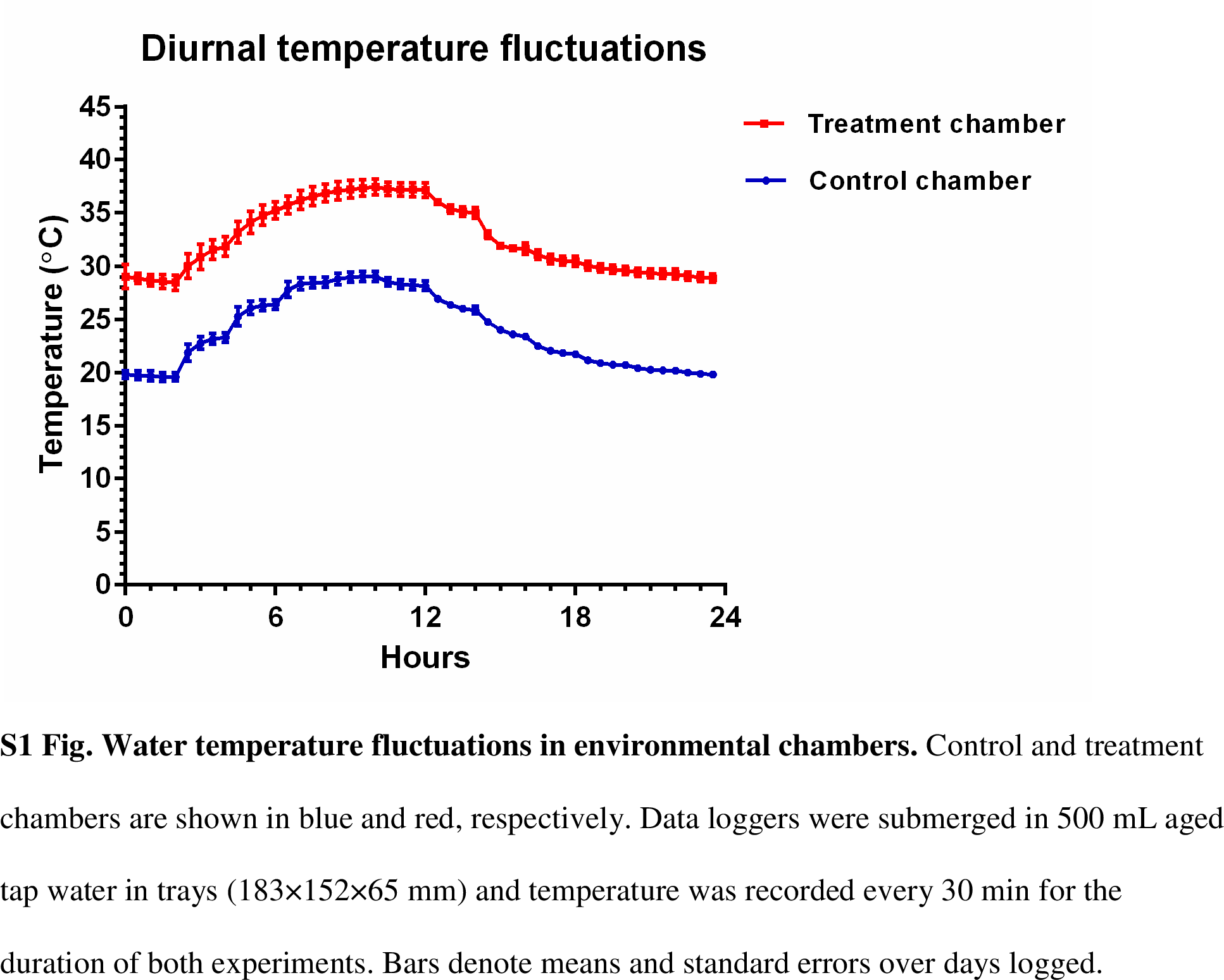

Supplement: S1 Fig — Control and treatment chambers are shown in blue and red, respectively. Data loggers were submerged in 500 mL aged tap water in trays (183 × 152 × 65 mm) and temperature was recorded every 30 min for the duration of both experiments. Bars denote means and standard errors over days logged. (TIF) [file pntd.0004873.s001.tif]

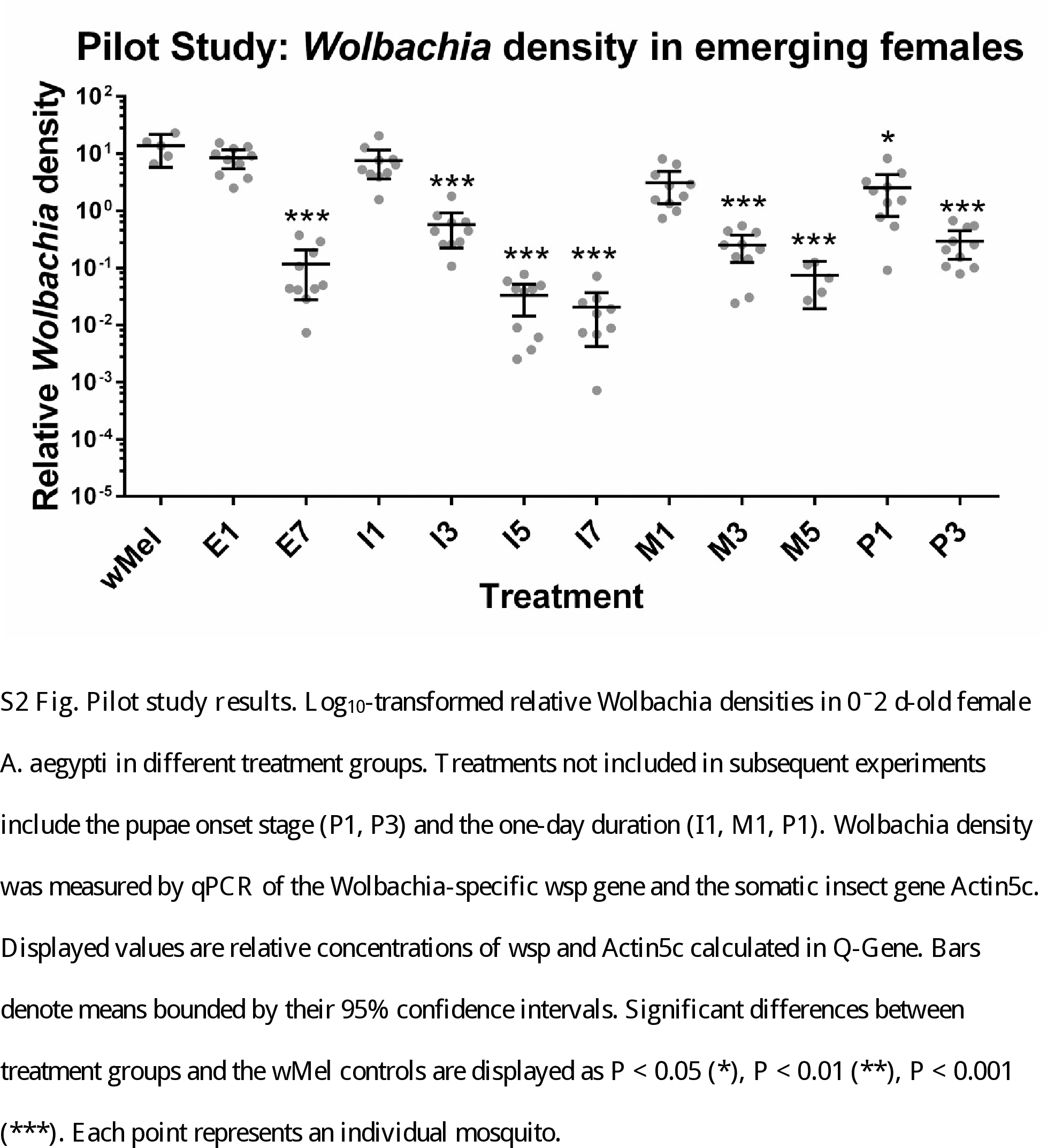

Supplement: S2 Fig — Log10-transformed relative Wolbachia densities in 0–2 d-old female A. aegypti in different treatment groups. Treatments not included in subsequent experiments include the pupae onset stage (P1, P3) and the one-day duration (I1, M1, P1). Wolbachia density was measured by qPCR of the Wolbachia-specific wsp gene and the somatic insect gene Actin5c. Displayed values are relative concentrations of wsp and Actin5c calculated in Q-Gene. Bars denote means bounded by their 95% confidence intervals. Significant differences between treatment groups and the wMel controls are displayed as P < 0.05 (*), P < 0.01 (**), P < 0.001 (***). Each point represents an individual mosquito. (TIF) [file pntd.0004873.s002.tif]

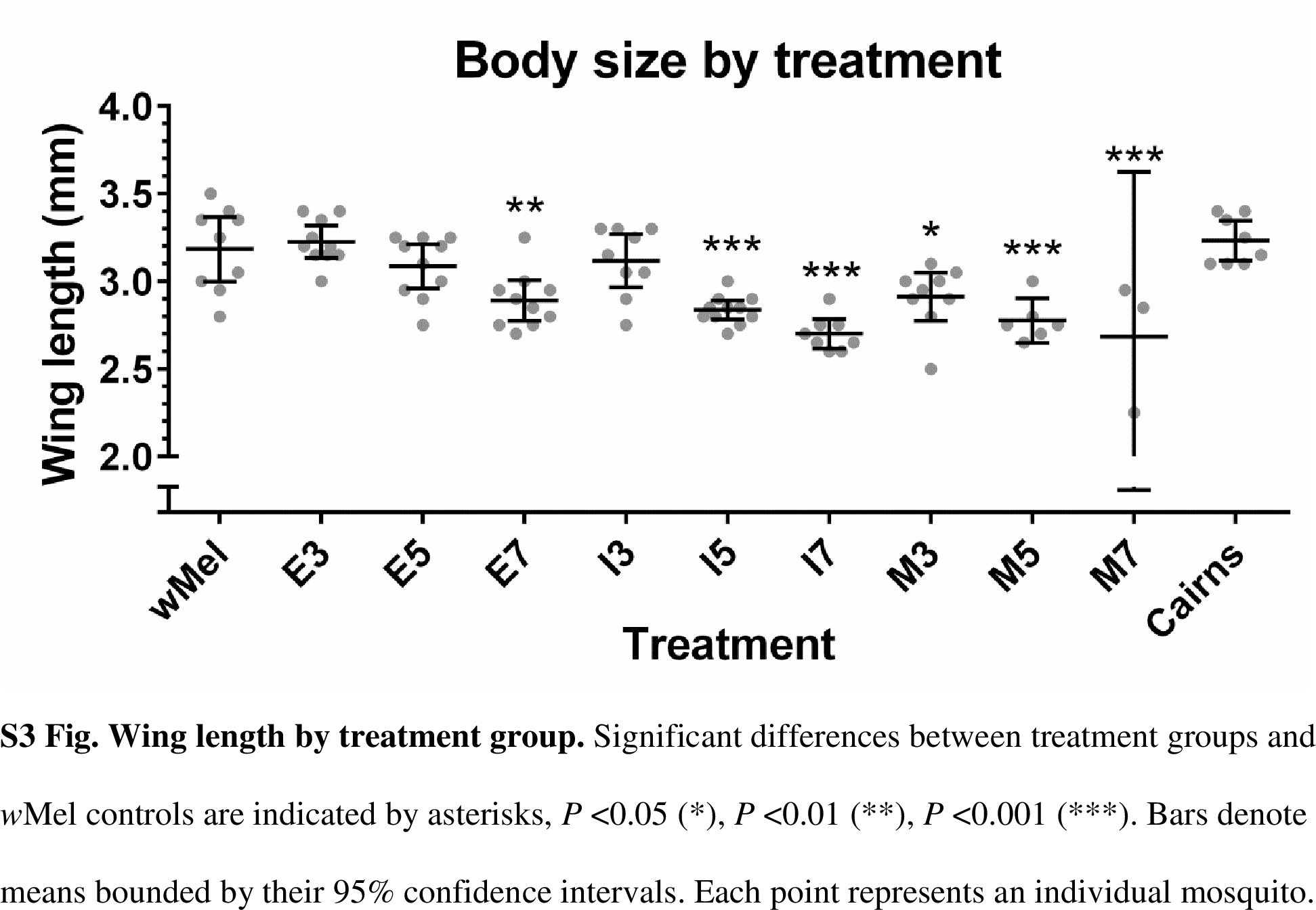

Supplement: S3 Fig — Significant differences between treatment groups and wMel controls are indicated by asterisks, P <0.05 (*), P <0.01 (**), P <0.001 (***). Bars denote means bounded by their 95% confidence intervals. Each point represents an individual mosquito. (TIF) [file pntd.0004873.s003.tif]

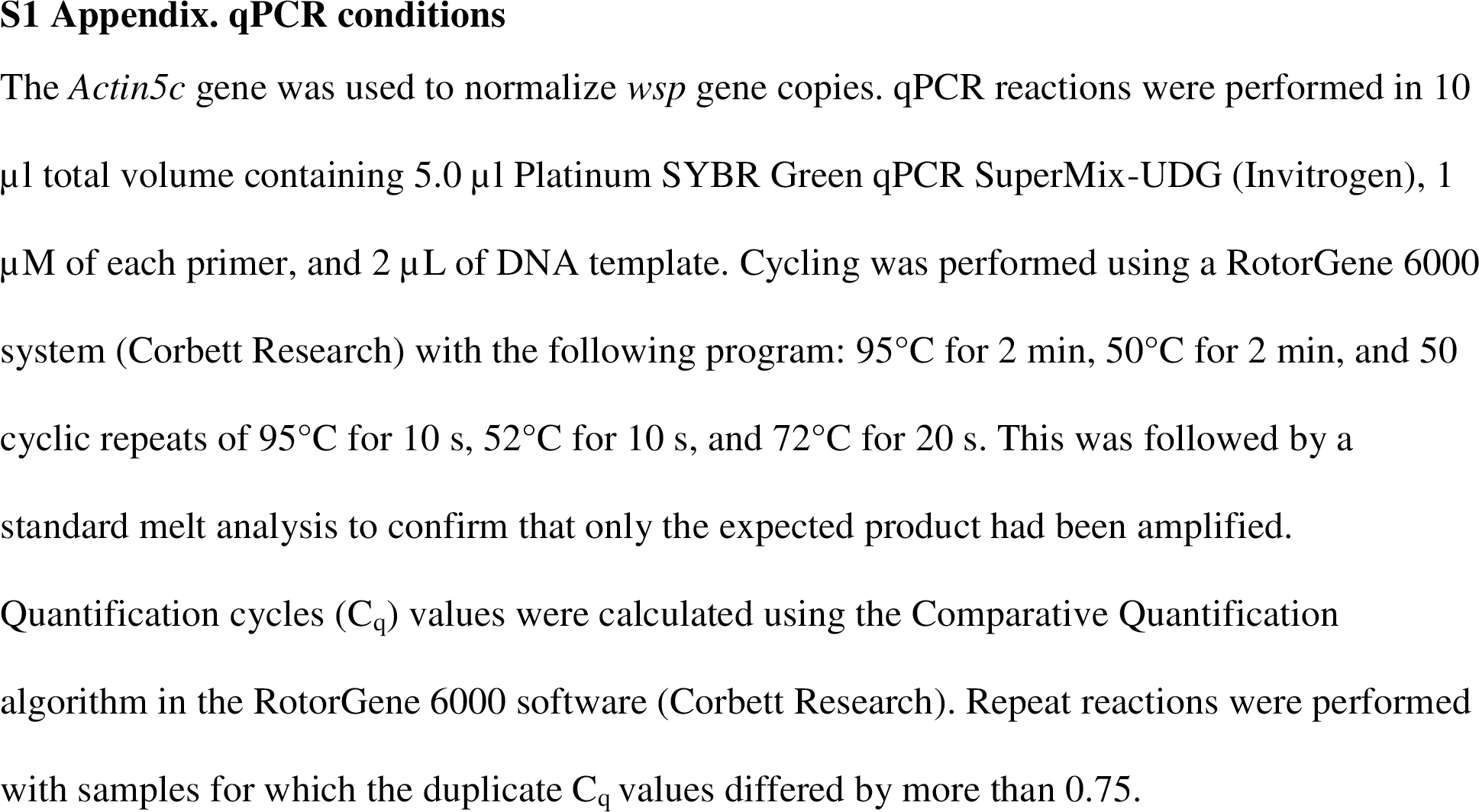

Supplement: S1 Appendix — The Actin5c gene was used to normalize wsp gene copies. qPCR reactions were performed in 10 μl total volume containing 5.0 μl Platinum SYBR Green qPCR SuperMix-UDG (Invitrogen), 1 μM of each primer, and 2 μL of DNA template. Cycling was performed using a RotorGene 6000 system (Corbett Research) with the following program: 95°C for 2 min, 50°C for 2 min, and 50 cyclic repeats of 95°C for 10 s, 52°C for 10 s, and 72°C for 20 s. This was followed by a standard melt analysis to confirm that only the expected product had been amplified. Quantification cycles (Cq) values were calculated using the Comparative Quantification algorithm in the RotorGene 6000 software (Corbett Research). Repeat reactions were performed with samples for which the duplicate Cq values differed by more than 0.75. (TIF) [file pntd.0004873.s004.tif]

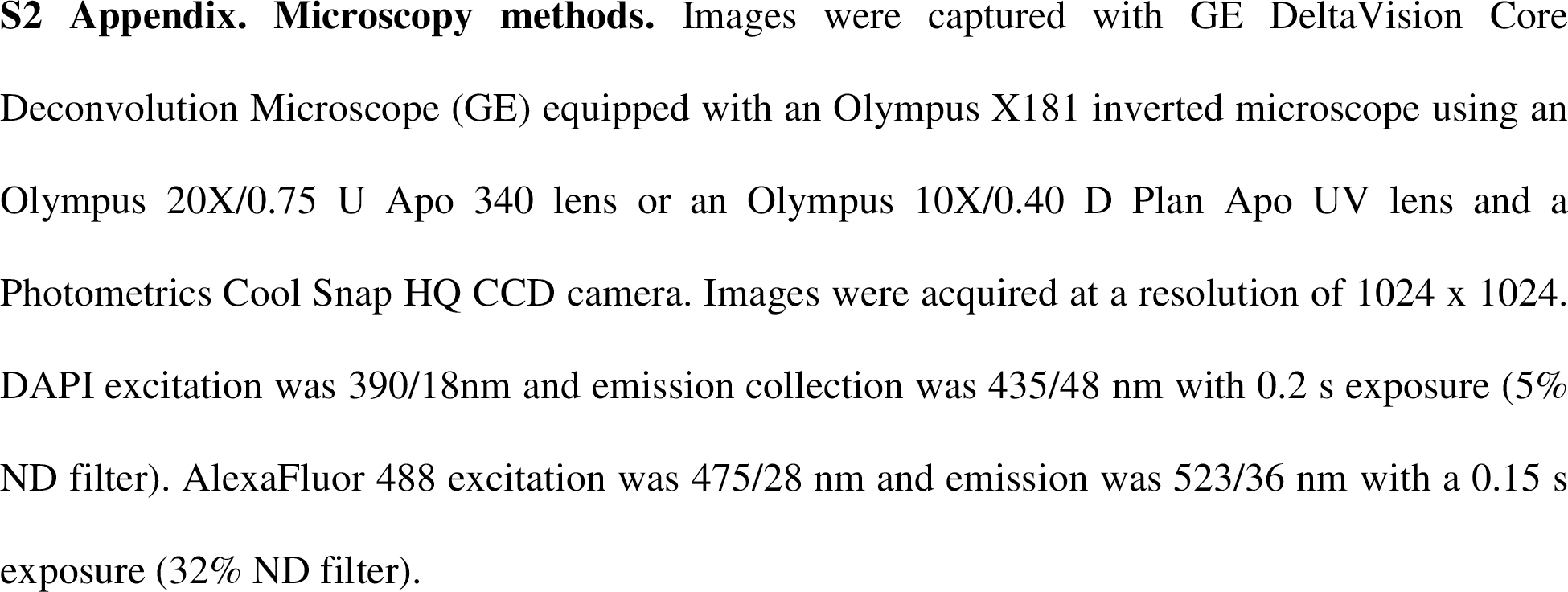

Supplement: S2 Appendix — Images were captured with GE DeltaVision Core Deconvolution Microscope (GE) equipped with an Olympus X181 inverted microscope using an Olympus 20X/0.75 U Apo 340 lens or an Olympus 10X/0.40 D Plan Apo UV lens and a Photometrics Cool Snap HQ CCD camera. Images were acquired at a resolution of 1024 x 1024. DAPI excitation was 390/18nm and emission collection was 435/48 nm with 0.2 s exposure (5% ND filter). AlexaFluor 488 excitation was 475/28 nm and emission was 523/36 nm with a 0.15 s exposure (32% ND filter). (TIF) [file pntd.0004873.s005.tif]
